# Supplementary material for: Spatio-temporal relays control layer identity of direction-selective neuron subtypes in Drosophila
Source: Nat Commun. 2018 Jun 12;9:2295. doi: 10.1038/s41467-018-04592-z (PMC5997761; doi:10.1038/s41467-018-04592-z)
Supplement: Supplementary file 1 — Supplementary Information [file 41467_2018_4592_MOESM1_ESM.pdf]

SUPPLEMENTARY INFORMATION

Spatio-temporal relays control layer identity of direction-selective neuron subtypes in *Drosophila*

Holger Apitz and Iris Salecker

SUPPLEMENTARY FIGURES

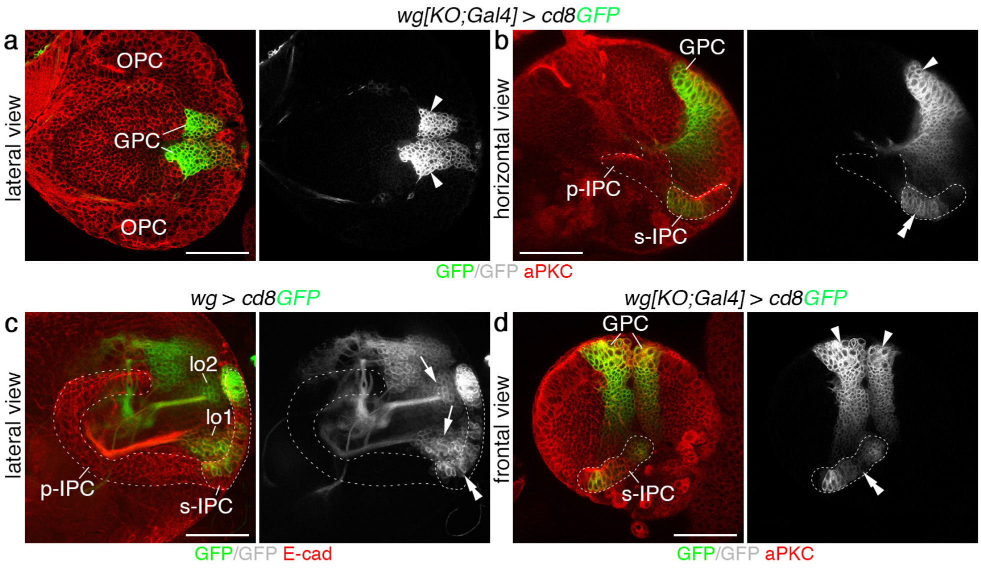

Supplementary Figure 1. Wild type *wg-Gal4* expression in the 3rd instar larval optic lobe. (**a**–**d**) *wg{KO;Gal4} UAS-cd8GFP* (**a**,**b**,**d**) and *wg-Gal4 UAS-cd8GFP* (**c**) (green) drive expression in the GPC areas (arrowheads) and the s-IPC (dashed line, double arrowheads). Expression is maintained in their progeny. Two lobula innervating neuron clusters (lo1 and lo2, arrows) originate from the s-IPC. No expression was observed in the p-IPC. Optic lobes are shown in lateral, horizontal and frontal orientations. For genotypes and sample numbers, see **Supplementary Table 2**. *Scale bars*, 50 μm.

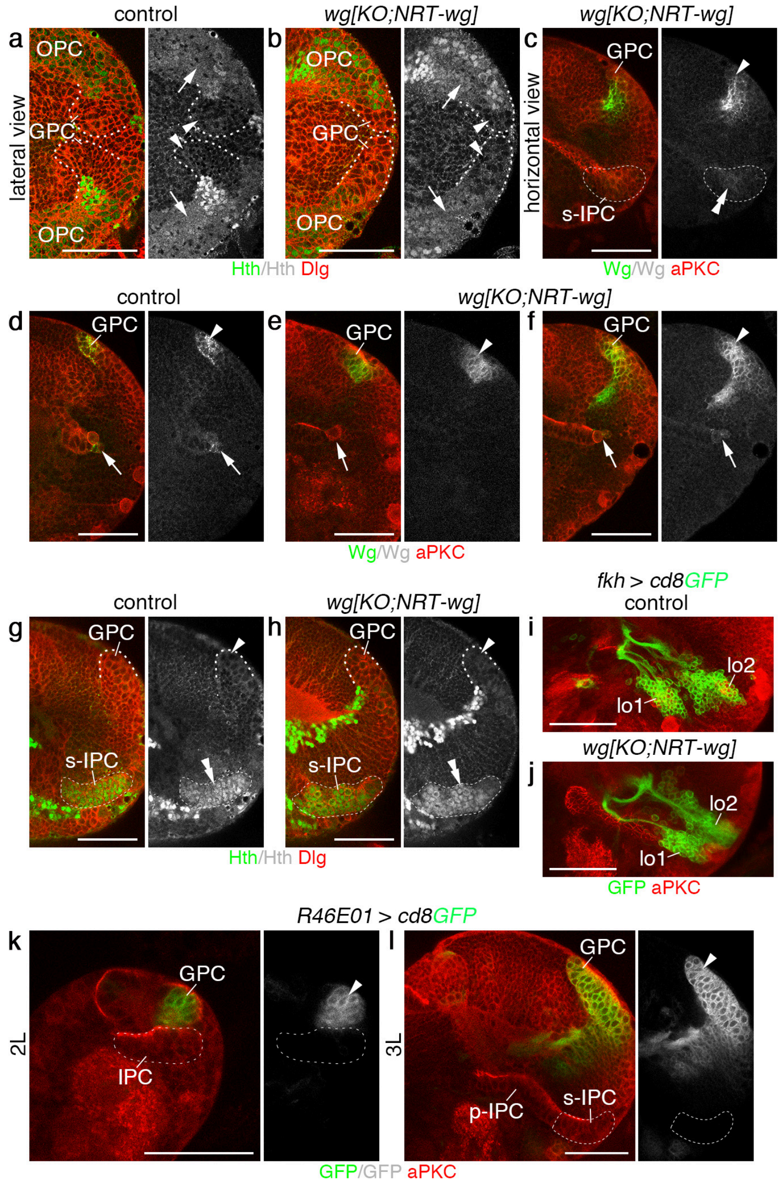

Supplementary Figure 2. Phenotypic analysis of *wg{KO;NRT-wg}* flies and GPC-specific *R46E01-Gal4* expression. Similar to controls (**a**), Hth (green) was expressed in the OPC (arrows) and suppressed in the GPC areas (arrowheads) of *wg{KO;NRT-wg}* flies (**b**). (**c**) In approximately 25% of samples, residual NRT-Wg (green) was detected in the s-IPC (double arrowheads) of *wg{KO;NRT-wg}* flies. Compared to controls (**d**), NRT-Wg (green) expression in the Nb clone (arrow) adjacent to the dorsal p-IPC arm was absent in approximately 75% (**e**) and present in approximately 25% (**f**) of *wg{KO;NRT-wg}* flies. Similar to controls (**g**), Hth (green) was expressed in the s-IPC (double arrowheads) of *wg{KO;NRT-wg}* flies (**h**). Similar to controls (**i**), the s-IPC in *wg{KO;NRT-wg}* flies generated two *fkh-Gal4 UAS-cd8GFP* (green) positive neuron clusters (lo1 and lo2) (**j**). *R46E01-Gal4 UAS-cd8GFP* expression (green) is specific to the GPC areas (arrowhead) and their progeny in 2nd (**k**) and late 3rd instar larvae (**l**). No labeling was detected in the IPC. For genotypes and sample numbers, see **Supplementary Table 2**. *Scale bars*, 50 μm.

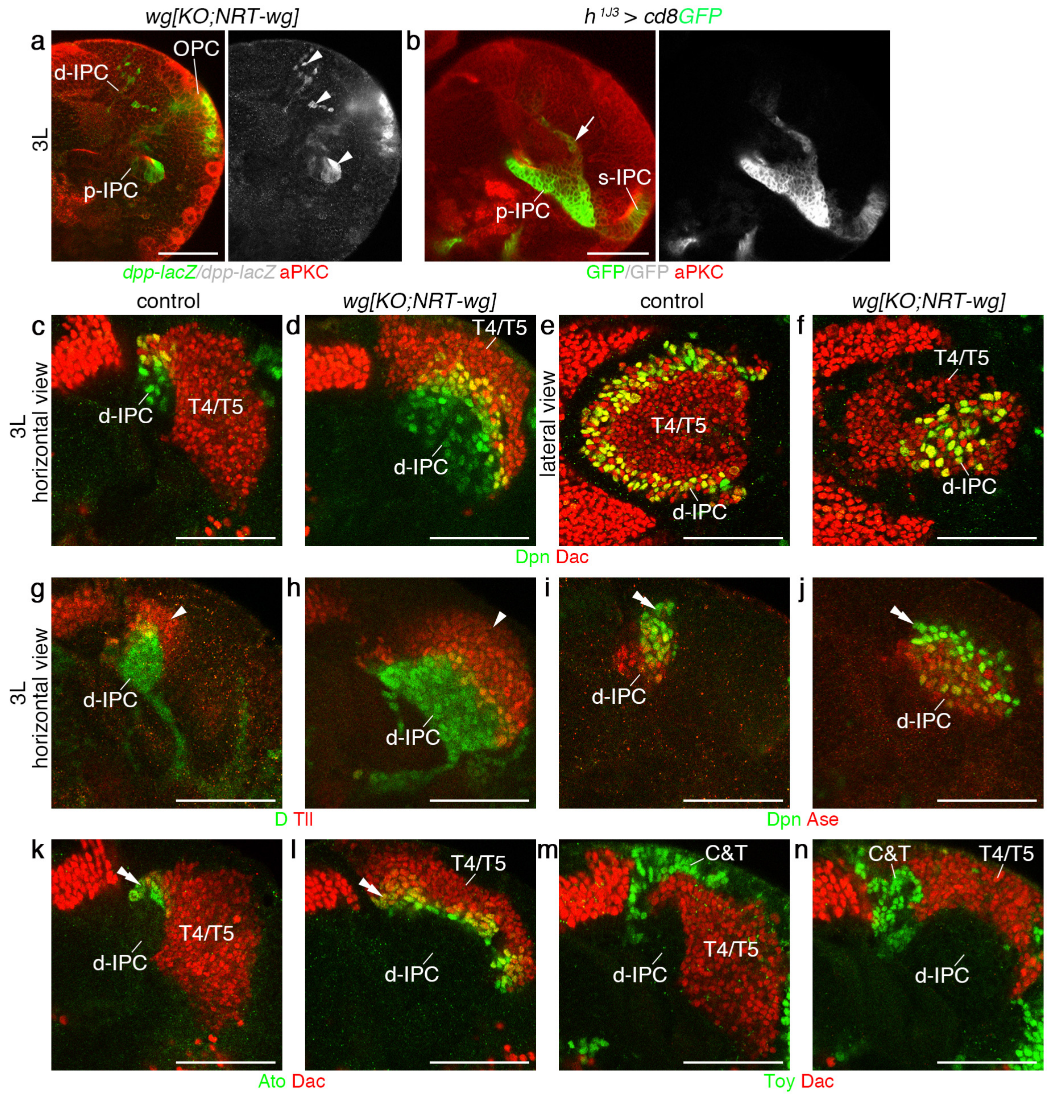

Supplementary Figure 3. Phenotypic analysis of *wg{KO;NRT-wg}* 3rd instar larval optic lobes and *h^{1J3}-Gal4* expression. (**a**) In approximately 50% of samples, residual *dpp-lacZ* expression (green, arrowheads) was observed in one IPC progenitor stream of *wg{KO;NRT-wg}* flies. (**b**) *h^{1J3}-Gal4 UAS-cd8GFP* (green) labeled the p-IPC, as well as progenitors (arrow) and the s-IPC. Similar to controls (**c**), Deadpan (Dpn, green) labeled d-IPC Nbs of *wg{KO;NRT-wg}* flies. Dac (red) was expressed in the second Nb competence window and maintained in T4/T5 neurons (**d**). However, the crescent-shaped d-IPC morphology (**e**) was altered in *wg{KO;NRT-wg}* flies (**f**). Similar to controls (**g**) in *wg{KO;NRT-wg}* flies (**h**), migratory progenitors and lower d-IPC Nbs expressed Dichaete (D, green) and upper d-IPC Nbs expressed Tailless (Tll, red). Tll was maintained in young T4/T5 neurons (arrowhead). Similar to controls (**i**), Ase (red) was expressed in lower d-IPC Nbs and GMCs and was absent from upper d-IPC Nbs labeled by Dpn (green, double arrowheads) in *wg{KO;NRT-wg}* flies (**j**). Similar to controls (**k**), Ato (green) and Dac (red) were expressed in upper d-IPC Nbs (double arrowheads) in *wg{KO;NRT-wg}* flies (**l**). Dac was maintained in T4/T5 neurons. Similar to controls (**m**), Toy (green) positive C&T neurons and Dac (red) positive T4/T5 neurons were generated in *wg{KO;NRT-wg}* flies (**n**). For genotypes and sample numbers, see **Supplementary Table 2**. *Scale bars*, 50 μm.

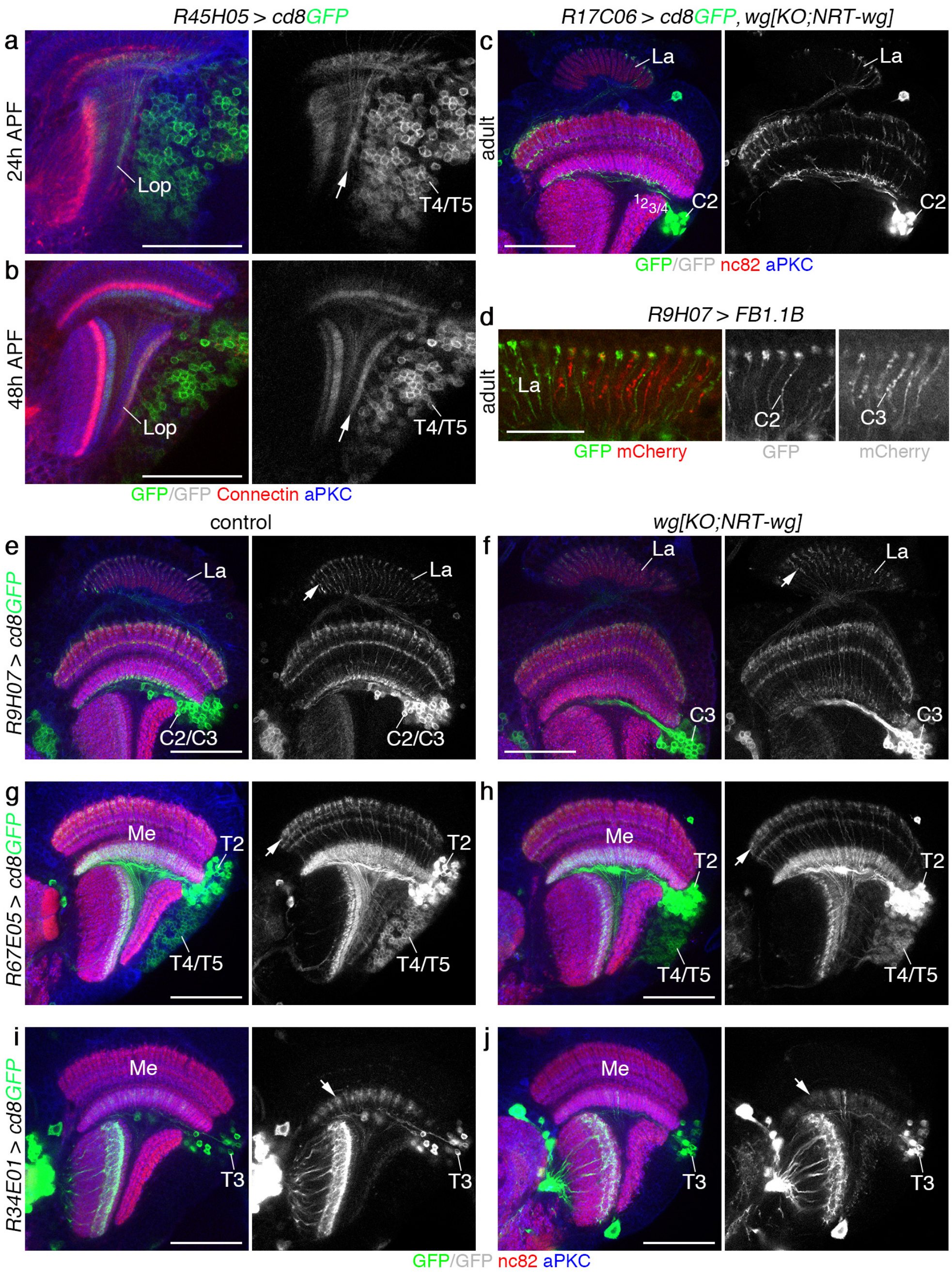

Supplementary Figure 4. *R45H05-Gal4 UAS-cd8GFP* expression in T4/T5 neurons innervating layers 3/4 and presence of C3 and T2/T3 neurons in *wg{KO;NRT-wg}* flies. *R45H05-Gal4 UAS-cd8GFP* (green) showed weak expression in T4/T5 neurons innervating lobula plate (Lop) layers 3/4 (arrow) at 24 h (**a**) and 48 h (**b**) after puparium formation (APF). (**c**) C2 neuron-specific *R17C06-Gal4 UAS-cd8GFP* (green) expression was partially absent in *wg{KO;NRT-wg}* flies with three lobula plate layers. (**d**) Flybow transgene expression using *R9H07-Gal4* labeled C2 and C3 neuron terminals in the lamina (La) with *GFP* (green) and *mCherry* (red). Similar to controls (**e**), C3 neurons labeled with *R9H07-Gal4 UAS-cd8GFP* (green) were present in *wg{KO;NRT-wg}* flies (**f**). Arrows highlight C3 neuron terminals in the lamina (La). Similar to controls (**g**), T2 neurons labeled with *R67E05-Gal4 UAS-cd8GFP* (green) were present in *wg{KO;NRT-wg}* flies (**h**). Arrows indicate T2 neurites in the medulla (Me). Similar to controls (**i**), T3 neurons labeled with *R34E01-Gal4 UAS-cd8GFP* (green) were present in *wg{KO;NRT-wg}* flies (**j**). Arrows indicate T3 neurites in the medulla (Me). For genotypes and sample numbers, see **Supplementary Table 2**. *Scale bars*, 50 μm (**a**–**c, e**–**j**), 25 μm (**d**).

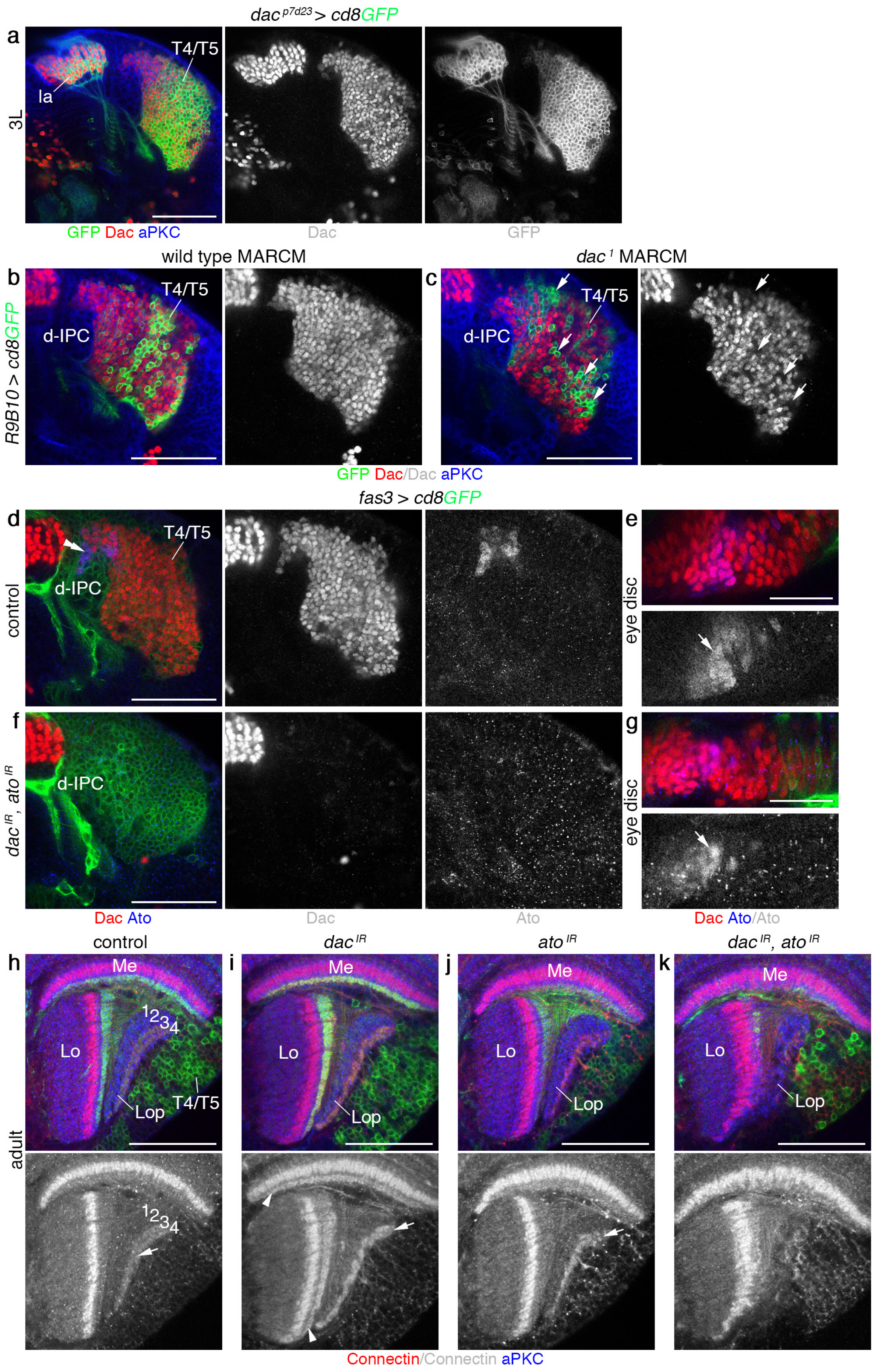

Supplementary Figure 5. Validation of *dac* reporter and mutant strains, and *dac* and *ato* RNAi lines. (**a**) *dac^{p7d23}-Gal4 UAS-cd8GFP* (green) faithfully reported Dac (red) expression in lamina and T4/T5 neurons in 3rd instar larval optic lobes. Unlike control neurons (**b**), *dac^{1}* mutant T4/T5 neurons (arrows) labeled with *R9B10-Gal4 UAS-cd8GFP* (green) did not express Dac (red) (**c**). In controls, Dac (red) and Ato (blue) were expressed in d-IPC Nbs during the second competence window (**d**). Dac was maintained in T4/T5 neurons. In the developing eye disc, Dac and Ato were co-expressed (**e**). IPC-specific

expression of $dac^{IR\ KK106040}$ and $ato^{IR\ TRiP.JF02089}$ using $fas3^{NP1233}$*-Gal4* resulted in efficient knockdown of Dac and Ato in the d-IPC and its progeny (**f**) without affecting expression in the developing eye disc (**g**). Unlike in controls (**h**), Connectin (red)-positive lobula plate (Lop) layers 3 and 4 (arrow) did not segregate following IPC-specific knockdown of *dac* (**i**) or *ato* (**j**) mediated by $fas3^{NP1233}$*-Gal4*. T4 and T5 dendrites (arrowheads) in the medulla (Me) and lobula (Lo) were labeled by Connectin following *dac* knockdown (**i**). Connectin-positive lobula plate layers were not observed following simultaneous *dac* and *ato* knockdown (**k**). For genotypes and sample numbers, see **Supplementary Table 2**. *Scale bars*, 50 µm (**a–d, f, h–k**), 25 µm (**e**,**g**).

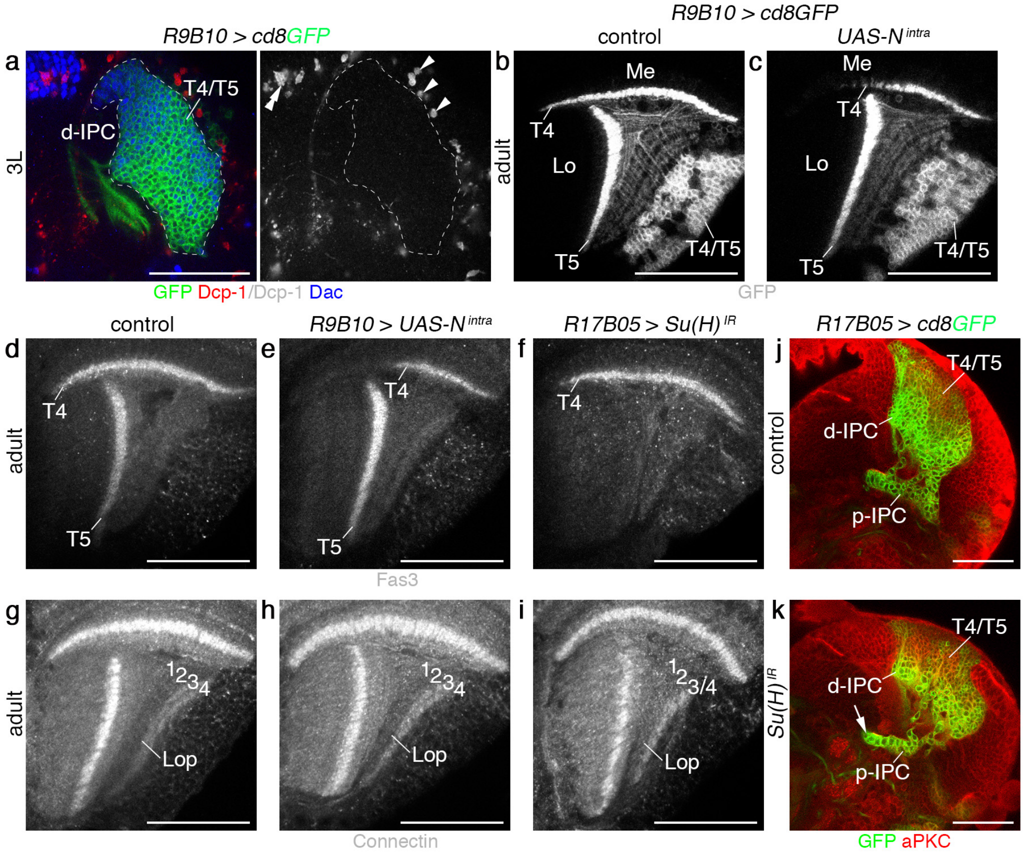

Supplementary Figure 6. Notch-dependent choice between T4 and T5 neuron fate. (**a**) Dcp-1 antibody (red) stained apoptotic cells inside the lamina (double arrowhead) and other areas (arrowheads) in 3rd instar larvae. It was not expressed in T4/T5 neurons (dotted line) labeled with *R9B10-Gal4 UAS-cd8GFP* (green) and Dac (blue). Unlike in controls (**b**), *R9B10-Gal4 UAS-cd8GFP* labeled T4 neurites were absent in the medulla (Me) following *UAS-*N^{intra} over-expression. In adults, only T4 neurons connecting to the anterior proximal medulla were affected (**c**). T5 neurites in the lobula (Lo) were unaffected. Fas3 labeling of T4/T5 dendrites in adults (**d**) confirmed that many T4 neurons were absent following *R9B10-Gal4 UAS-*N^{intra} over-expression (**e**). T5 neurons were absent following *R17B05-Gal4* mediated IPC-specific knockdown of *Su(H)* (**f**). While Connectin was expressed - similar to controls (**g**) - in lobula plate (Lop) layers 3/4 of *R9B10-Gal4 UAS-*N^{intra} flies (**h**), layers were not segregated following *R17B05-Gal4* mediated IPC-specific knockdown of *Su(H)* (**i**). Compared to controls (**j**), p-IPC morphology (arrow) was distorted following *R17B05-Gal4 UAS-cd8GFP* (green) mediated knockdown of *Su(H)* in the IPC of 3rd instar larvae (**k**). For genotypes and sample numbers, see **Supplementary Table 2**. *Scale bars*, 50 µm.

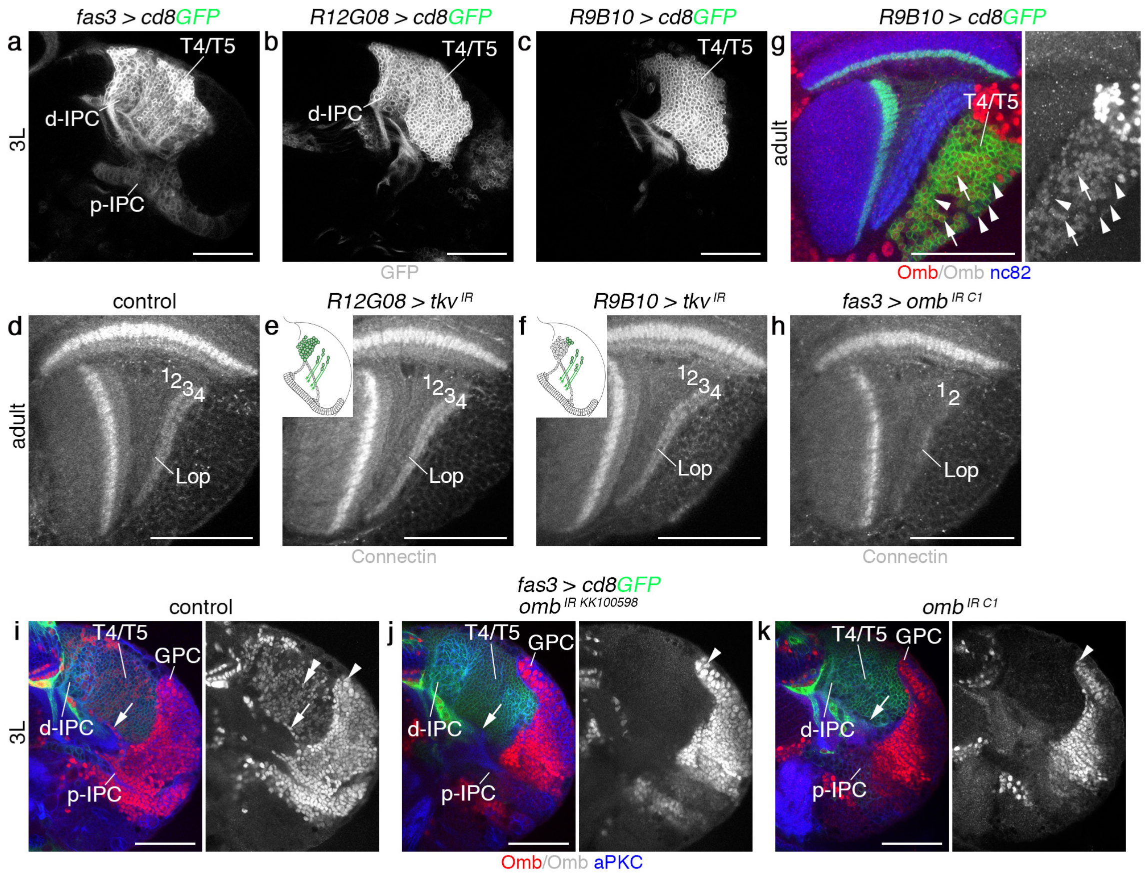

Supplementary Figure 7. p-IPC-specific requirement of *tkv* and validation of *omb* RNAi lines. *fas3*^{NP1233}-*Gal4* drives *UAS-cd8GFP* expression in the whole IPC and its progeny (**a**). *R12G08-Gal4* expression was restricted to the d-IPC and progeny (**b**), and *R9B10-Gal4* to second Nb competence window in the d-IPC and T4/T5 neurons (**c**). Similar to controls (**d**), *tkv* knockdown in the d-IPC and/or T4/T5 neurons using *R12G08-Gal4* (**e**) and *R9B10-Gal4* (**f**) did not affect lobula plate (Lop) layers 3/4 labeled with Connectin. (**g**) Omb expression (red, arrows) in T4/T5 neuron subsets was maintained to adulthood. Arrowheads indicate Omb-negative *R9B10-Gal4 UAS-cd8GFP* labeled T4/T5 neurons (green). (**h**) Lobula plate (Lop) layer 3/4-specific Connectin labeling was absent in adults following IPC-specific knockdown of *omb*$^{IR\ C1}$ using *fas3*^{NP1233}-*Gal4*. Unlike in controls (**i**), IPC-specific expression of *omb*$^{IR\ KK100598}$ (**j**) and *omb*$^{IR\ C1}$ (**k**) using *fas3*^{NP1233}-*Gal4* resulted in efficient knockdown of Omb (red) in the p-IPC, progenitors (arrows), d-IPC and T4/T5 neurons (double arrowheads). The GPC areas (arrowhead) and progeny were not affected. For genotypes and sample numbers, see **Supplementary Table 2**. *Scale bars*, 50 μm.

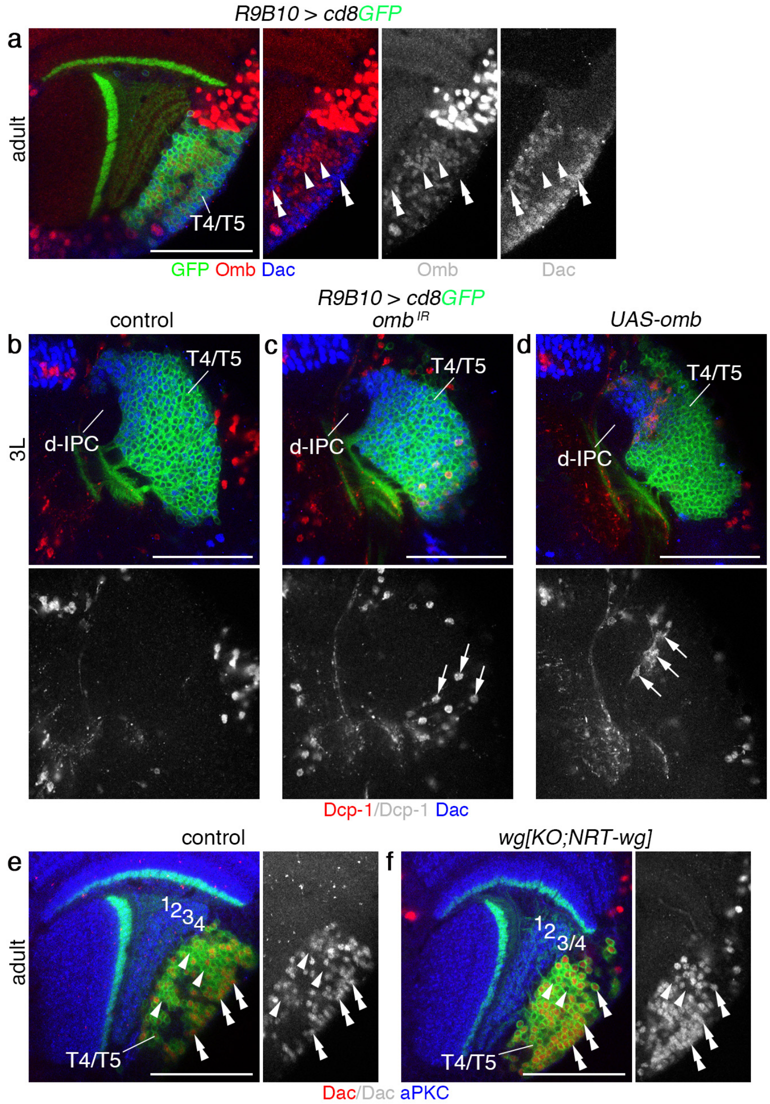

Supplementary Figure 8. Omb and Dac expression in adults and *omb* knockdown- and overexpression-mediated apoptosis of T4/T5 neurons. (**a**) Omb (red, arrowheads) and Dac (blue, double arrowheads) showed mutually exclusive expression in *R9B10-Gal4 UAS-cd8GFP* (green) labeled T4/T5 neurons in adults. Unlike in controls (**b**), *R9B10-Gal4 UAS-cd8GFP* (green) mediated *omb* knockdown (**c**) and over-expression (**d**) resulted in Dcp-1 (red) labeled apoptotic T4/T5 neurons (arrows) in 3rd instar larvae. Compared to controls (**e**), *wg{KO;NRT-wg}* flies with three lobula plate layers (**f**) had similar Dac (red) positive (double arrowheads), but less Dac-negative (arrowheads) T4/T5 neurons labeled with *R9B10-Gal4 UAS-cd8GFP* (green). For genotypes and sample numbers, see **Supplementary Table 2**. *Scale bars*, 50 μm.

SUPPLEMENTARY TABLES

Supplementary Table 1. Full genotypes and sample numbers shown in Figures 1-8.

Figure	Panel	Genotype	n = [a]
Fig. 1	d	UAS-FB1.1B^{260b}/+; R9B10-Gal4/+	11
	e	UAS-FB1.1B^{260b}/+; R9B10-Gal4/+	13
	f	wg[KO;NRT-wg]/wg[KO;NRT-wg]; R9B10-Gal4/UAS-FB1.1B^{49b}	18/30
	g	wg[KO;NRT-wg]/wg[KO;NRT-wg]; R9B10-Gal4/UAS-FB1.1B^{49b}	12/30
	i	UAS-FB1.1B^{260b}/+; R9B10-Gal4/+	26
	j	wg[KO;NRT-wg]/wg[KO;NRT-wg]; R9B10-Gal4/UAS-FB1.1B^{49b}	16/30
	k	wg[KO;NRT-wg]/wg[KO;NRT-wg]; R9B10-Gal4/UAS-FB1.1B^{49b}	14/30
	l-n	UAS-FB1.1B^{260b}/+; R46E01-Gal4/+	10
Fig. 2	a	w^{1118}; wg[KO;NRT-wg]/+	11
	b	wg[KO;NRT-wg]/wg[KO;NRT-wg]	30/41
	c	UAS-FB1.1B^{260b}/+; R46E01-Gal4/+	6
	d	wg[KO;Gal4]/CyO; UAS-HRP-cd8GFP/TM2	18
	e	wg[KO;Gal4]/CyO; UAS-HRP-cd8GFP/TM2	12
	f	wg[KO;NRT-wg]/wg[KO;Gal4]; UAS-HRP-cd8GFP/+	6
	g	wg[KO;>wg^{+}> NRT-wg] UAS-NRT-wg/wg[KO;Gal4]; UAS-FLP/tubP-Gal80^{ts}	16
	h	wg[KO;>wg^{+}>NRT-wg] UAS-NRT-wg/wg[KO;Gal4]; UAS-FLP/tubP-Gal80^{ts}	26/34
	i	wg[KO;>NRT-wg>wg^{+}]/wg[KO;NRT-wg]; UAS-FLP/+	11
	j	wg[KO;>NRT-wg>wg^{+}]/wg[KO;NRT-wg]; UAS-FLP/R46E01-Gal4	16/23
	k	fz3^{G00357}-GFP/w^{1118} or Y	11
	l	wg[KO;Gal4]/+; UAS-HRP-cd8GFP/notum^{WRE}-lacZ	10
	m	w^{1118}	7
	n	ey$^{3.5}$-Gal80/+ or Y; fas3^{NP1233}-Gal4/UAS-fz2$^{IR\ KK108998}$; UAS-Dcr2 UAS-cd8GFP/UAS-fz$^{IR\ GD43077}$	14/17
Fig. 3	a, b, g	wg[KO;Gal4]/+; UAS-HRP-cd8GFP/dpp-lacZ$^{Exel.2}$	47
	c, d	wg-Gal4/+; UAS-cd8GFP/dpp-lacZ$^{Exel.2}$	11
	e	wg[KO;Gal4]/+; UAS-HRP-cd8GFP/dpp-lacZ$^{Exel.2}$	8
	h	wg[KO;NRT-wg]/wg[KO;NRT-wg]; dpp-lacZ$^{Exel.2}$/+	16/32[b]
	i	UAS-arm^{s10}/UAS-cd8GFP; dpp-lacZ$^{Exel.2}$/h^{1J3}-Gal4	9
	j	ey$^{3.5}$-Gal80/+ or Y; fas3^{NP1233}-Gal4/UAS-wg$^{IR\ GD13351}$; UAS-Dcr2 UAS-cd8GFP/dpp-lacZ$^{Exel.2}$	7
	k	ey$^{3.5}$-Gal80/+ or Y; fas3^{NP1233}-Gal4/UAS-tkv$^{IR\ KK105834}$; UAS-Dcr2 UAS-cd8GFP/dpp-lacZ$^{Exel.2}$	11
	l	ey$^{3.5}$-Gal80/w ^{1118} or Y; fas^{NP1233}-Gal4/+; UAS-Dcr2 UAS-cd8GFP/+	8
	m	ey$^{3.5}$-Gal80/+ or Y; fas3^{NP1233}-Gal4/UAS-fz2$^{IR\ KK108998}$; UAS-Dcr2 UAS-cd8GFP/UAS-fz$^{IR\ GD43077}$	5
	n	ey$^{3.5}$-Gal80/+ or Y; fas3^{NP1233}-Gal4/UAS-tkv$^{IR\ KK105834}$; UAS-Dcr2 UAS-cd8GFP/+	7
Fig. 4	a	UAS-cd8GFP/+; R45H05-Gal4/dpp-lacZ$^{Exel.2}$	7
	b	UAS-FB1.1B^{260b}/+; R45H05-Gal4/+	5
	c, e	act>y^{+}>Gal4 UAS-GFP/tubP-Gal80^{ts}; UAS-FLP/R45H05-Gal4	8
	d	UAS-FB1.1B^{260b}/hs-mFLP5^{MH12}; R9B10-Gal4/+	26
	f	UAS-FB1.1B^{260b}/+; R17C06-Gal4/+	4
	g	wg[KO;NRT-wg]/wg[KO;NRT-wg]; R17C06-Gal4/UAS-FB1.1B^{49b}	7/9[c]
Fig. 5	b	UAS-FB1.1B^{260b}/+; R9B10-Gal4/+	6
	c	UAS-FB1.1B^{260b}/+; R9B10-Gal4/+	6
	d	UAS-FB1.1B^{260b}/dac^{p7d23}-Gal4	4
	e	UAS-FB1.1B^{260b}/dac^{p7d23}-Gal4	4

	g	yw hs-FLP^{122}; tubP-Gal80^{LL10} FRT40A/FRT40A; UAS-FB1.1B^{49b} R9B10-Gal4/+	13
	h	yw hs-FLP^{122}; tubP-Gal80^{LL10} FRT40A/dac^{1} FRT40A; UAS-FB1.1B^{49b} R9B10-Gal4/+	14
	i	yw hs-FLP^{122}; tubP-Gal80^{LL10} FRT40A/ FRT40A; UAS-FB1.1B^{49b} R9B10-Gal4/UAS-brp-RFP	5
	j	yw hs-FLP^{122}; tubP-Gal80^{LL10} FRT40A/dac^{1} FRT40A; UAS-FB1.1B^{49b} R9B10-Gal4/UAS-brp-RFP	11/14
	k	ey$^{3.5}$-Gal80/w^{1118} or Y; fas^{NP1233}-Gal4/+; UAS-Dcr2 UAS-cd8GFP/+	4
	l	ey$^{3.5}$-Gal80/+ or Y; fas3^{NP1233}-Gal4/UAS-dac$^{IR\ KK106040}$; UAS-Dcr2 UAS-cd8GFP/UAS-ato$^{IR\ TRiP.JF02089}$	4
	m	ey$^{3.5}$-Gal80/w^{1118} or Y; fas^{NP1233}-Gal4/+; UAS-Dcr2 UAS-cd8GFP/+	3
	n	ey$^{3.5}$-Gal80/+ or Y; fas3^{NP1233}-Gal4/UAS-dac$^{IR\ KK106040}$; UAS-Dcr2 UAS-cd8GFP/UAS-ato$^{IR\ TRiP.JF02089}$	6
Fig. 6	a	UAS-FB1.1B^{260b}/+; R9B10-Gal4/+	6
	b	UAS-FB1.1B^{260b}/UAS-N^{intra}; R9B10-Gal4/+	4
	c	R17B05-Gal4 UAS-FB1.1C^{49b}/+	7
	d	UAS-Su(H)$^{IR\ KK103597}$/+; UAS-Dcr2 UAS-cd8GFP/R17B05-Gal4 UAS-FB1.1C^{49b}	15
Fig. 7	a	UAS-cd8GFP/UAS-FB1.1B^{260b}; dpp^{blk1}-Gal4/+	12
	b	UAS-cd8GFP/+; dpp^{blk1}-Gal4/+	24
	d	omb^{P1}-lacZ/+ or Y; UAS-FB1.1B^{260b}/+; R9B10-Gal4/+	5
	e	omb^{P1}-lacZ/+ or Y; wg[KO;NRT-wg]/wg[KO;NRT-wg]	6/10[d]
	f	ey$^{3.5}$-Gal80/w^{1118} or Y; fas3^{NP1233}-Gal4/+; UAS-Dcr2 UAS-cd8GFP/+	7
	g	ey$^{3.5}$-Gal80/+ or Y; fas3^{NP1233}-Gal4/UAS-tkv$^{IR\ KK105834}$; UAS-Dcr2 UAS-cd8GFP/+	8
	h	wg[KO;Gal4]/+; UAS-HRP-cd8GFP/dpp-lacZ$^{Exel.2}$	47
	i	ey$^{3.5}$-Gal80/+ or Y; fas3^{NP1233}-Gal4/UAS-omb$^{IR\ KK100598}$; UAS-Dcr2 UAS-cd8GFP/dpp-lacZ$^{Exel.2}$	25
	j	UAS-FB1.1B^{260b}/+; R9B10-Gal4/+	11
	k	ey$^{3.5}$-Gal80/+ or Y; fas3^{NP1233}-Gal4/UAS-omb$^{IR\ KK100598}$; UAS-Dcr2 UAS-cd8GFP/+	10
	l	UAS-cd8GFP/UAS-omb$^{IR\ KK100598}$; R12G08-Gal4/UAS-Dcr2 UAS-cd8GFP	6
	m	UAS-FB1.1B^{260b}/UAS-omb$^{IR\ KK100598}$; R9B10-Gal4/UAS-Dcr2 UAS-cd8GFP	8
Fig. 8	a	UAS-FB1.1B^{260b}/+; R9B10-Gal4/+	8
	b	UAS-FB1.1B ^{260b}/+; R9B10-Gal4/+	7
	c	UAS-FB1.1B ^{260b}/+; R9B10-Gal4/+	6
	d	UAS-FB1.1B ^{260b}/UAS-omb$^{IR\ KK100598}$; R9B10-Gal4/UAS-Dcr2 UAS-cd8GFP	5
	e	UAS-FB1.1B^{260b}/+; R9B10-Gal4/UAS-omb	6
	f	wg[KO;NRT-wg]/wg[KO;NRT-wg]; R9B10-Gal4/UAS-FB1.1B^{49b}	5
	g	wg[KO;NRT-wg]/wg[KO;NRT-wg]; R9B10-Gal4 UAS-FB1.1B^{49b}/UAS-omb	5
	i	UAS-FB1.1B^{260b}/+; R9B10-Gal4/+	11
	j	UAS-FB1.1B^{260b}/+; R9B10-Gal4/UAS-omb	8
	k	wg[KO;NRT-wg]/wg[KO;NRT-wg]; R9B10-Gal4 UAS-FB1.1B^{49b}/UAS-omb	16

> indicate *FRT* sites; to facilitate the understanding of experiments, *UAS-FB1.1B* is described as *cd8GFP* in figures;

[a] If not otherwise indicated, in loss-of-function and knockdown experiments, all examined control samples were normal, while all experimental samples showed defects (100% penetrance);

[b] *dpp-lacZ* expression in the IPC was absent (n=16/32) or showed residual labeling in one progenitor stream (n=16/32);

[c] C2 neurons were fully (n=7/9) or partially (n=2/9) absent in samples with two or three lobula plate layers, respectively;

[d] *omb*^{P1}*-lacZ* expression was absent (n=6/10) or showed residual labeling (n=4/10) in the IPC.

Supplementary Table 2. Full genotypes and sample numbers shown in Supplementary Figures 1-8.

Figure	Panel	Genotype	***n* =** [a]
Fig. S1	a, b, d	*wg[KO;Gal4]/+; UAS-HRP-cd8GFP/dpp-lacZ*$^{Exel.2}$	47
	c	*wg-Gal4/+; UAS-cd8GFP/dpp-lacZ*$^{Exel.2}$	11
Fig. S2	a, g	*wg[KO;Gal4]/+; UAS-HRP-cd8GFP/+*	3
	b, h	*wg[KO;NRT-wg]/wg[KO;NRT-wg]*	5
	c	*wg[KO;NRT-wg]/wg[KO;NRT-wg]*	11/41
	d	*w*^{1118}	5
	e	*wg[KO;NRT-wg]/wg[KO;NRT-wg]*	31/41
	f	*wg[KO;NRT-wg]/wg[KO;NRT-wg]*	10/41
	i	*UAS-FB1.1B*^{260b}*/+; fkh-Gal4/+*	4
	j	*wg[KO;NRT-wg]/wg[KO;NRT-wg]; fkh-Gal4/UAS-FB1.1B*^{49b}	6
	k	*UAS-FB1.1B*^{260b}*/+; R46E01-Gal4/+*	6
	l	*UAS-FB1.1B*^{260b}*/+; R46E01-Gal4/+*	10
Fig. S3	a	*wg[KO;NRT-wg]/wg[KO;NRT-wg]; dpp-lacZ*$^{Exel.2}$*/+*	16/32[b]
	b	*UAS-cd8GFP/+; h*^{1J3}*-Gal4/dpp-lacZ*$^{Exel.2}$	3
	c, e	*UAS-FB1.1B*^{260b}*/+; R9B10-Gal4/+*	3
	d, f	*wg[KO;NRT-wg]/wg[KO;NRT-wg]; R9B10-Gal4/UAS-FB1.1B*^{49b}	4
	g	*w*^{1118}	6
	h	*wg[KO;NRT-wg]/wg[KO;NRT-wg]; R9B10-Gal4/UAS-FB1.1B*^{49b}	6
	i	*w*^{1118}	7
	j	*wg[KO;NRT-wg]/wg[KO;NRT-wg]; R9B10-Gal4/UAS-FB1.1B*^{49b}	7
	k	*w*^{1118}	12
	l	*wg[KO;NRT-wg]/wg[KO;NRT-wg]*	8
	m	*UAS-FB1.1B*^{260b}*/+; R9B10-Gal4/+*	3
	n	*wg[KO;NRT-wg]/wg[KO;NRT-wg]; R9B10-Gal4/UAS-FB1.1B*^{49b}	5
Fig. S4	a	*UAS-FB1.1B*^{260b}*/+; R45H05-Gal4/+*	6
	b	*UAS-FB1.1B*^{260b}*/+; R45H05-Gal4/+*	3
	c	*wg[KO;NRT-wg]/wg[KO;NRT-wg]; R17C06-Gal4/UAS-FB1.1B*^{49b}	2/9[c]
	d	*UAS-FB1.1B*^{260b}*/hs-mFLP5*^{MH12}*; R9H07-Gal4/+*	4
	e	*UAS-FB1.1B*^{260b}*/+; R9H07-Gal4/+*	4
	f	*wg[KO;NRT-wg]/wg[KO;NRT-wg]; R9H07-Gal4/UAS-FB1.1B*^{49b}	8
	g	*UAS-FB1.1B*^{260b}*/+; R67E05-Gal4/+*	6
	h	*wg[KO;NRT-wg]/wg[KO;NRT-wg]; R67E05-Gal4/UAS-FB1.1B*^{49b}	9
	i	*UAS-FB1.1B*^{260b}*/+; R34E01-Gal4/+*	4
	j	*wg[KO;NRT-wg]/wg[KO;NRT-wg]; R34E01-Gal4/UAS-FB1.1B*^{49b}	11
Fig. S5	a	*UAS-FB1.1B*^{260b}*/dac*^{p7d23}*-Gal4*	4
	b	*yw hs-FLP*^{122}*; tubP-Gal80*^{LL10} *FRT40A/ FRT40A; UAS-FB1.1B*^{49b} *R9B10-Gal4/+*	7
	c	*yw hs-FLP*^{122}*; tubP-Gal80*^{LL10} *FRT40A/ dac*^{1} *FRT40A; UAS-FB1.1B*^{49b} *R9B10-Gal4/+*	7
	d, e	*ey*$^{3.5}$*-Gal80/w*^{1118} or *Y*; *fas*^{NP1233}*-Gal4/+*; *UAS-Dcr2 UAS-cd8GFP/+*	4
	f, g	*ey*$^{3.5}$*-Gal80/+* or *Y*; *fas3*^{NP1233}*-Gal4/UAS-dac*$^{IR\ KK106040}$; *UAS-Dcr2 UAS-cd8GFP/UAS-ato*$^{IR\ TRiP.JF02089}$	8
	h	*ey*$^{3.5}$*-Gal80/w*^{1118} or *Y*; *fas*^{NP1233}*-Gal4/+*; *UAS-Dcr2 UAS-cd8GFP/+*	8
	i	*ey*$^{3.5}$*-Gal80/+* or *Y*; *fas3*^{NP1233}*-Gal4/UAS-dac*$^{IR\ KK106040}$; *UAS-Dcr2 UAS-cd8GFP/+*	6
	j	*ey*$^{3.5}$*-Gal80/+* or *Y*; *fas3*^{NP1233}*-Gal4/+*; *UAS-Dcr2 UAS-cd8GFP/UAS-ato*$^{IR\ TRiP.JF02089}$	6
	k	*ey*$^{3.5}$*-Gal80/+* or *Y*; *fas3*^{NP1233}*-Gal4/UAS-dac*$^{IR\ KK106040}$; *UAS-Dcr2 UAS-cd8GFP/UAS-ato*$^{IR\ TRiP.JF02089}$	7

Fig. S6	a	*UAS-FB1.1B*^{260b}*/+; R9B10-Gal4/+*	5
	b	*UAS-FB1.1B*^{260b}*/+; R9B10-Gal4/+*	13
	c	*UAS-FB1.1B*^{260b}*/UAS-N*^{intra}*; R9B10-Gal4/+*	11
	d	*UAS-FB1.1B*^{260b}*/+; R9B10-Gal4/+*	3
	e	*UAS-FB1.1B*^{260b}*/UAS-N*^{intra}*; R9B10-Gal4/+*	8
	f	*UAS-Su(H)*$^{IR\ KK103597}$*/+; UAS-Dcr2 UAS-cd8GFP/R17B05-Gal4 UAS-FB1.1C*^{49b}	10
	g	*UAS-FB1.1B*^{260b}*/+; R17B05-Gal4/+*	4
	h	*UAS-FB1.1B*^{260b}*/UAS-N*^{intra}*; R9B10-Gal4/+*	8
	i	*UAS-Su(H)*$^{IR\ KK103597}$*/+; UAS-Dcr2 UAS-cd8GFP/R17B05-Gal4 UAS-FB1.1C*^{49b}	11
	j	*UAS-FB1.1B*^{260b}*/+; R17B05-Gal4/+*	7
	k	*UAS-Su(H)*$^{IR\ KK103597}$*/+; UAS-Dcr2 UAS-cd8GFP/R17B05-Gal4 UAS-FB1.1C*^{49b}	5
Fig. S7	a	*ey*$^{3.5}$*-Gal80/w*^{1118} or *Y*; *fas*^{NP1233}*-Gal4/UAS-FB1.1B*^{260b}; *UAS-Dcr2 UAS-cd8GFP/+*	4
	b	*UAS-FB1.1B*^{260b}*/UAS-cd8GFP; R12G08-Gal4/+*	4
	c	*UAS-FB1.1B*^{260b}*/+; R9B10-Gal4/+*	4
	d	*UAS-FB1.1B*^{260b}*/+; R9B10-Gal4/+*	11
	e	*UAS-cd8GFP/UAS-tkv*$^{IR\ KK105834}$*; R12G08-Gal4/UAS-Dcr2 UAS-cd8GFP*	13
	f	*UAS-FB1.1B*^{260b}*/UAS-tkv*$^{IR\ KK105834}$*; R9B10-Gal4/UAS-Dcr2 UAS-cd8GFP*	6
	g	*UAS-FB1.1B*^{260b}*/+; R9B10-Gal4/+*	3
	h	*ey*$^{3.5}$*-Gal80*/+ or *Y*; *fas3*^{NP1233}*-Gal4/UAS-omb*$^{IR\ C1}$; *UAS-Dcr2 UAS-cd8GFP/+*	7
	i	*ey*$^{3.5}$*-Gal80/w*^{1118} or *Y*; *fas*^{NP1233}*-Gal4*/+; *UAS-Dcr2 UAS-cd8GFP/+*	5
	j	*ey*$^{3.5}$*-Gal80*/+ or *Y*; *fas3*^{NP1233}*-Gal4/UAS-omb*$^{IR\ KK100598}$; *UAS-Dcr2 UAS-cd8GFP/+*	6
	k	*ey*$^{3.5}$*-Gal80*/+ or *Y*; *fas3*^{NP1233}*-Gal4/UAS-omb*$^{IR\ C1}$; *UAS-Dcr2 UAS-cd8GFP/+*	5
Fig. S8	a	*UAS-FB1.1B*^{260b}*/+; R9B10-Gal4/+*	6
	b	*UAS-FB1.1B*^{260b}*/+; R9B10-Gal4/+*	5
	c	*UAS-FB1.1B*^{260b}*/UAS-omb*$^{IR\ KK100598}$*; R9B10-Gal4/UAS-Dcr2 UAS-cd8GFP*	5
	d	*UAS-FB1.1B*^{260b}*/+; R9B10-Gal4/UAS-omb*	5
	e	*UAS-FB1.1B*^{260b}*/+; R9B10-Gal4/+*	6
	f	*wg[KO;NRT-wg]/wg[KO;NRT-wg]; R9B10-Gal4/UAS-FB1.1B*^{49b}	5

> indicate *FRT* sites; to facilitate the understanding of experiments, *UAS-FB1.1B* is described as *cd8GFP* in figures.

[a] If not otherwise indicated, in loss-of-function and knockdown experiments, all examined control samples were normal, while all experimental samples showed defects (100% penetrance);

[b] *dpp-lacZ* expression showed residual labeling in one progenitor stream (n=16/32) or was absent in the IPC (n=16/32);

[c] C2 neurons were fully (n=7/9) or partially (n=2/9) absent in samples with two or three lobula plate layers, respectively.
