# Supplementary material for: Spatio-temporal relays control layer identity of direction-selective neuron subtypes in Drosophila
Source: Nat Commun. 2018 Jun 12;9:2295. doi: 10.1038/s41467-018-04592-z (PMC5997761; doi:10.1038/s41467-018-04592-z)
Supplement: Supplementary file 3 — Description of Additional Supplementary Files [file 41467_2018_4592_MOESM3_ESM.pdf]

**Description of Additional Supplementary Files**

**File Name:** Supplementary Data 1
**Description:** Supplementary Data 1 contain the source data used for quantifications shown in Figs. 1h and 8h.
